# The emerging role of long non‐coding RNA in spinal cord injury

**DOI:** 10.1111/jcmm.13515

**Published:** 2018-02-01

**Authors:** Zhongju Shi, Bin Pan, Shiqing Feng

**Affiliations:** ^1^ Department of Orthopaedics Tianjin Medical University General Hospital Tianjin China; ^2^ Department of Orthopaedics the Affiliated Hospital of Xuzhou Medical University Xuzhou Jiangsu China

**Keywords:** spinal cord injury, long non‐coding RNA, neuron, astrocyte, oligodendrocyte, microglia, inflammation, angiogenesis

## Abstract

Spinal cord injury (SCI) is a significant health burden worldwide which causes permanent neurological deficits, and there are approximately 17,000 new cases each year. However, there are no effective and current treatments that lead to functional recovery because of the limited understanding of the pathogenic mechanism of SCI. In recent years, the biological roles of long non‐coding RNAs (lncRNAs) in SCI have attracted great attention from the researchers all over the world, and an increasing number of studies have investigated the regulatory roles of lncRNAs in SCI. In this review, we summarized the biogenesis, classification and function of lncRNAs and focused on the investigations on the roles of lncRNAs involved in the pathogenic processes of SCI, including neuronal loss, astrocyte proliferation and activation, demyelination, microglia activation, inflammatory reaction and angiogenesis. This review will help understand the molecular mechanisms of SCI and facilitate the potential use of lncRNAs as diagnostic markers and therapeutic targets for SCI treatment.

## Introduction

Spinal cord injury (SCI) is a severe trauma often caused by car accidents or falls, and it is characteristic of high incidence, high morbidity and huge cost 1. SCI is an overwhelming neurological disorder and have devastating physiological consequences, and it affects approximately 180,000 new patients each year 2, 3. SCI comprises primary and secondary phases 4. After the initial trauma in spinal cord caused by a bone fracture or compression, SCI can lead to many complex pathological changes including inflammatory responses, hypoxia and neuronal cell death 5, 6. Because of the inhabitation microenvironment after SCI, the regenerative capacity of the adult spinal cord is poor, and it can result in severe sensory and motor deficits 2. Therefore, understanding the pathophysiological and biological processes involved in SCI is essential to promote functional recovery in patients with SCI.

Long non‐coding RNAs (lncRNAs) are defined as thousands of RNA transcripts longer than 200 nucleotides in length with no protein‐coding potential, and lncRNAs have attracted great attention from the researchers all over the world 7, 8. LncRNAs can be grouped into five main categories: long intergenic non‐coding RNAs (lincRNAs), intronic lncRNAs, antisense lncRNAs, divergent lncRNAs and enhancer‐derived lncRNAs 9, 10, 11. LncRNAs can be detected in the nucleus, cytoplasm or both; however, they are was mainly located in cell nucleus 12, 13. It has been shown that lncRNAs could regulate the downstream genes expression *via* mediating chromatin, transcriptional and post‐transcriptional modification, and lncRNAs have been regarded as a new frontier in the study of many human diseases 14, 15, 16. The roles of lncRNAs in cell physiology include chromatin remodelling, transcriptional and epigenetic regulatory factors, RNA conformational dynamics, modification of specific protein targets and control of protein complex formation and localization 17. However, for the vast majority of lncRNAs, their action and physiological function in spinal cord injury remain to be uncovered.

In this review, we overviewed the recent studies on the molecular functions of lncRNAs involving in spinal cord injury, and further discussed their potential roles of diagnosis and prognostic biomarkers and therapeutic targets, and this review aimed to help deepen the current understanding of lncRNAs in spinal cord injury.

## The biogenesis, classification and function of lncRNAs

Non‐coding RNAs (ncRNAs) cover more than 98% of the human genome 18. LncRNAs are a subgroup of non‐coding RNA transcripts longer than 200 nucleotides, most of lncRNAs are transcribed by RNA polymerase II and are capped at the 5′ end, spliced and polyadenylated 19, 20, 21. LncRNAs were initially thought to be ‘transcriptional noise’ of the transcriptome 22. In recent years, the research of lncRNAs developed rapidly, and lncRNAs were being increasingly recognized as key regulators of many cellular processes, especially gene expression 23, 24, 25. Compared to mRNAs, lncRNAs are generally regarded to be exquisitely regulated and are restricted to specific cell types 26.

According to their location in the genome, lncRNAs can be classified into five categories: (*i*) sense lncRNAs, lncRNAs overlap with one or more exons from another transcript in the same strand; (*ii*) antisense lncRNAs, lncRNAs overlap with one or more exons from another transcript in the opposite strand; (*iii*) bidirectional lncRNAs, lncRNAs that share their promoter with another gene in the opposite strand and are initiated <1000 base pairs away in close genomic proximity; (*iv*) intronic lncRNAs, lncRNAs initiated completely within an intron of a protein‐coding gene without overlapping exons; and (*v*) intergenic lncRNAs (also termed large intervening non‐coding RNAs or lincRNAs), those that are independent transcripts located between two genes 27, 28, 29.

The main functions of lncRNAs are as follows: (*i*) scaffold molecules for providing stabilization in chromatin modification complexes; (*ii*) providing molecular guides for localization of their binding targets; (*iii*) as signals, regulated the expression of target genes by recognizing the transcription factors; (*iv*) as decoys, binding to molecules and block their role on their target genes; (*v*) acting as miRNA ‘sponges’ to regulate mRNA activity by sharing common miRNA‐binding sites with mRNAs; and (*vi*) acting as enhancer RNAs or even encoding short peptides with regulating function 24, 26, 27, 30, 31, 32. However, there is still biological significance of a large number of lncRNAs remaining unclear and needing to further illuminated.

## Alterations and regulation in lncRNAs expression following SCI

After SCI, a series of pathophysiological events occur at molecular and cellular levels. To understand the changes in the expression levels and the corresponding regulatory functions of lncRNAs following SCI, previous studies have performed microarray method and RNA sequencing and found that a large number of lncRNAs are found in the spinal cord and changed following SCI 33, 34, 35. Wang *et al*. performed a large‐scale screening of expression changes of lncRNAs in a rat contusion SCI model at 1, 4 and 7 days following SCI, and the transcripts with a false discovery rate (FDR) ≤0.001 and a fold change ≥2 were considered as differentially expressed 35. Wang *et al*. demonstrated that seven lncRNAs that were detected in the adult rat spinal cord showed significant expressional changes, and among the seven lncRNAs, two (LOC100910973, H19) were up‐regulated, one (RGD1559747) was down‐regulated, four (Rn28s, Rn45s, RT1‐CE6, Rmrp) were up‐regulated at 1 day post‐SCI, and then subsequently down‐regulated at 4 and 7 days post‐SCI 35. Ding *et al*. used a microarray method in a contusion SCI mouse model at 1 day, 3 days, 1 weeks and 7 weeks following SCI, and the results showed that few changes were found at 1 day following SCI and the changes peaked 1 week following SCI and subsequently decreased compared with sham operation group 33. Another study investigates alterations of the lncRNAs expression of in the subchronic and chronic stages of SCI (1 month, 3 months and 6 months post‐SCI) using RNA sequencing, and the analyses of the SCI transcriptome identified 277 differentially expressed lncRNAs 34. LncRNAs microarray and lncRNA sequencing studies involving SCI are summarized in Table 1. These researches on lncRNAs provided new molecular information and have shown that the changes of lncRNAs expression have effects on many key processes of SCI physiopathology. Thus, these researches may provide novel insights into the molecular mechanisms of SCI.

**Table 1:** Summary of studies about the changes of lncRNAs expression following spinal cord injury

References	SCI model				Methods	LncRNA expression changes
	Animal	Method	Level	Sampling		
35	Adult female SD rats (200–250 g)	Contusive spinal cord injury using an NYU impactor (10 g, 12.5 mm)	T10	1, 4 and 7 days post‐SCI	RNA sequencing	1 dpo: 1 down, 6 up 4 dpo: 5 down, 2 up 7 dpo: 5 down, 2 up
33	Male ICR mice (20–25 g) aged 6–8 weeks	Multicenter Animal Spinal Cord Injury Study (MASCIS) Impactor weight‐drop device(5 g, 25 mm)	T10	1 day, 3 days, 1 week and 3 weeks post‐SCI	Microarray	1 dpo: 181 down, 164 up 3 dpo: 290 down, 212 up 1 wpo: 565 down, 326 up 3 wpo: 40 down, 141 up
34	Adult female SD rats aged 12–14 weeks	Moderate contusion injury	T9	1, 3 and 6 months post‐SCI	RNA sequencing	1mpo: 17 down, 120 up 3mpo: 77 down, 162 up 6mpo: 54 down, 125 up

SD rats: Sprague–Dawley rats; ICR mice: Institute of Cancer Research mice; T: thoracic level; SCI: spinal cord injury; dpo: days post‐operation; wpo: weeks post‐operation; mpo: months post‐operation.

## The regulative roles of lncRNAs in neural cells behaviour

### Neuron

After SCI, permanent neuronal loss is a major obstacle and will result in functional disfigurement, so enhancing the survival of neurons is critical for recovery in patients with SCI 36. It is really difficult to avoid the neuronal loss happened; however, during the secondary injury after SCI, genes expression can be regulated to promote post‐traumatic spinal neuron survival, and regulation of lncRNAs is an important way 37, 38. LncRNAs have been found to function in the central nervous system (CNS) development and neurogenesis 39, 40. Many exploratory studies characterizing lncRNA expression involving neuron behaviour. In one study, the researchers demonstrate that lncRNA‐Map2k4 can promote neuron proliferation and inhibit neuron apoptosis through a miR‐199a/FGF1 pathway 41. LncRNA IGF2AS was first identified as a cancer regulator in Wilm's tumours, a recent study showed that inhibiting endogenous lncRNA IGF2AS can promote neuronal growth and protect local anaesthetic‐induced neurotoxicity in DRG neurons 42. Pnky is a neural‐specific lncRNA that regulates neurogenesis, and a previous study showed that knockdown of Pnky may promote neuronal differentiation 40, and this result is beneficial to improve the results of neural stem cells transplantation in patients with SCI. Together, these studies have provided a good start for understanding the roles of lncRNAs in neuron behaviour, and in‐depth analysis in this field is very necessary.

### Astrocyte

In the central nervous system, astrocytes are the most abundant glial cell type 43, 44. Astrocytes have been a primary focus of researchers in both neuropathology and neurophysiology, and they play an essential role in provision of energy metabolites to neurons and maintenance of the extracellular balance of ions 45, 46. As a typical feature following SCI, astrocyte proliferation and reactive gliosis can contribute to the formation of glial scar and lead to a physical and biochemical barrier to plasticity and regeneration, and inhibit functional recovery finally 47, 48. However, reactive astrocytes also serve as beneficial factors for SCI including endogenous neuroprotection and secreting growth‐promoting neurotrophic factors 43. Therefore, reactive astrocytes can be beneficial or detrimental for SCI, so how to make full use of its positive aspect and inhibit its detrimental aspects will be the future directions of research. More recent studies have begun to examine the effects of lncRNAs on astrocyte proliferation and reactive gliosis. In a previous study, the researchers knocked down lncSCIR1 expression in cultured astrocytes and found that down‐regulation of lncSCIR1 may promote astrocyte proliferation and migration *in vitro* and might play a detrimental role in the pathophysiology of SCI 35. Another study showed that lncRNA Gm4419 could promote trauma‐induced astrocyte apoptosis *via* up‐regulating the expression of inflammatory cytokine tumour necrosis factor α (TNF‐α), and the up‐regulation of TNF‐α was possible *via* competitively binding miR‐466 l 49. Therefore, identifying the crucial lncRNAs to regulate the astrocyte proliferation and activation has become the main concern in SCI treatment.

### Oligodendrocyte

Myelin sheath, the insulating layer surrounding the axon, is necessary for maintaining structural and functional integrity of neural circuits in vertebrate spinal cords 50, 51. Apoptosis of myelin‐forming oligodendrocytes (OLs) and demyelination of surviving axons is a vital part of the cascading secondary events in spinal cord injury (SCI), and then it leads to conduction failure 52, 53. Furthermore, the post‐injury microenvironment can limit the endogenous oligodendrogenesis and remyelination processes *via* increased remyelination‐inhibitory molecules 54. Therefore, enhance remyelination is one of the important factors in promoting functional recovery in SCI. In recent years, more and more researchers found that lncRNAs may be promising therapeutic targets for spinal cord repair, so it is necessary to know the roles of lncRNAs in remyelination. He *et al*. established dynamic expression profiles of lncRNAs at different development stages of oligodendrocyte, and they found that overexpression of lncOL1 promotes the differentiation of precocious oligodendrocyte in the developing brain and inactivation of lncOL1 could cause the defects in CNS myelination and remyelination after injury 55. Furthermore, lncOL1 could promote oligodendrocyte maturation *via* interaction with Suz12 55. In another study, the researchers identified lncRNAs that are regulated during Oligodendrocyte Precursor Cell (OPC) differentiation from Neural Stem Cells (NSCs), and they found that lnc‐OPC was the top candidate and showed highly specific expression in OPCs 56. This study elucidated the roles of lncRNAs in OPC fate determination 56. Further researches involving the effect of lncRNAs on remyelination are needed to provide useful clues for the treatment of SCI.

### Microglia

Inflammation is a crucial biological process in response to injury, infection and trauma suffered by cells or tissues, and it involves the cells present within the central nervous system (CNS), including the neurons, macroglia and microglia 57. Microglia are known as the resident macrophages of CNS, which play a key role in active immune defence mechanism in the CNS, and they are also a type of glial cell lesser in number than astrocytes 58, 59. Activated microglia can release many pro‐inflammatory molecules, such as interleukin‐1beta (IL‐1β), TNF‐α, reactive oxygen species and nitric oxide 60. After SCI, microglia undergo significant cellular, molecular and functional changes, and microglial activation is often used to represent neuronal inflammation during secondary phases of SCI 61, 62. A recent study demonstrated that lncRNA fantom3_F730004F19 may be associated with microglia‐induced inflammation *via* the Toll‐like receptor signalling pathway in early brain injury (EBI) following subarachnoid haemorrhage (SAH) 63. Qi *et al*. demonstrated that lncRNA SNHG14 could increase the expression of PLA2G4A by inhibition of miR‐145‐5p, which resulted in the activation of microglia 64. Activated microglia can be divided into two functional types: M1/classic and M2/alternative polarization 65. M1 polarized microglia have generally been considered to promote neuronal apoptosis and inhibit OPCs differentiation into mature OLs, whereas M2 polarized microglia are known to promote neuronal survival, neurite outgrowth and OPCs differentiation 66, 67, 68. In a recent study, the researchers identified the lncRNA GAS5 as an epigenetic regulator of microglial polarization and suggested that GAS5 may be a promising target for the treatment of demyelinating diseases 69. Furthermore, the function of lncRNAs in activation of microglia following SCI also requires further study.

## LncRNAs involved in inflammation after SCI

After SCI, the inflammatory response involves the activation of microglia and the infiltration of neutrophils, monocytes and lymphocytes 70. As mentioned above, microglia are capable of regulating activation and polarization of microglia, so what are the roles of lncRNAs in infiltration of neutrophils, monocytes and lymphocytes? As a type of inflammatory cell, neutrophils are the main cells and enter the injury site first 71. It has been shown that many lncRNAs are present in neutrophils and the levels of lncRNA expression are associated with development, differentiation and activation of neutrophils 72, 73. Furthermore, previous studies have identified lncRNAs expressed in B lymphocytes and T lymphocytes development and activation 74, 75. Panzeri *et al*. suggested that long intergenic non‐coding RNAs could be novel drivers of human lymphocyte differentiation 76. Moreover, previous researches performed RNA sequencing of monocytes from four individuals and combined their data with eleven other publicly available datasets and provided a landscape of lncRNAs in monocytes 77. LncRNA LINC00341 has been shown to suppress vascular cell adhesion molecule 1 (VCAM1) expression and inhibit monocyte adhesion, and it also has the anti‐inflammatory effect 78. A recent research demonstrated that lncRNA LINC00305 could promote monocyte inflammation *via* activating the AHRR‐NF‐κB pathway 79. Although various publications report lncRNAs to regulate inflammation after SCI, most of the related mechanism of lncRNAs is not fully understood. Therefore, further experiments are needed to confirm lncRNAs to be essential in the inflammation process.

## LncRNAs involved in angiogenesis after SCI

After SCI, injury to vessels may lead to haemorrhage 80. An important cause of cell and tissue damage in the injured spinal cord is the ischaemic conditions brought about by injury to blood vessels following SCI 81, 82. Therefore, it is a problem that how to promote angiogenesis and provide the transport of oxygen, growth factors and other nutrients to the injured spinal cord 83. Accumulating evidence showed that lncRNAs could directly regulate the process of angiogenesis by targeting related signalling molecules 84, 85, 86, 87. A recent study showed that lncRNA HIF1A‐AS2 could promote the angiogenesis in human umbilical vein endothelial cells (HUVECs) in hypoxia *via* facilitating the up‐regulation of HIF‐1α by sponging to miR‐153‐3p 88. LncRNA MEG3 is known as an important tumour suppressor in some human cancers, and overexpression of MEG3 suppressed the angiogenesis in vascular endothelial cells (VECs) significantly 89. Furthermore, MEG3 was significantly decreased after ischaemic stroke, and overexpression of Meg3 could decrease the capillary density after ischaemic stroke *via* inhibition of notch signalling 90. Thus, MEG3 may play an important role in the control of angiogenesis following SCI. Moreover, previous studies demonstrated that two intergenic lncRNAs called linc00323‐003 and MIR503HG are induced by hypoxia in endothelial cells, which would help promote angiogenesis 91. Overall, more and more evidence indicates that lncRNA plays an important role in angiogenesis; however, the role of these lncRNAs in angiogenesis following SCI need to be examined in future research.

## Conclusion and perspective

**Figure 1:**
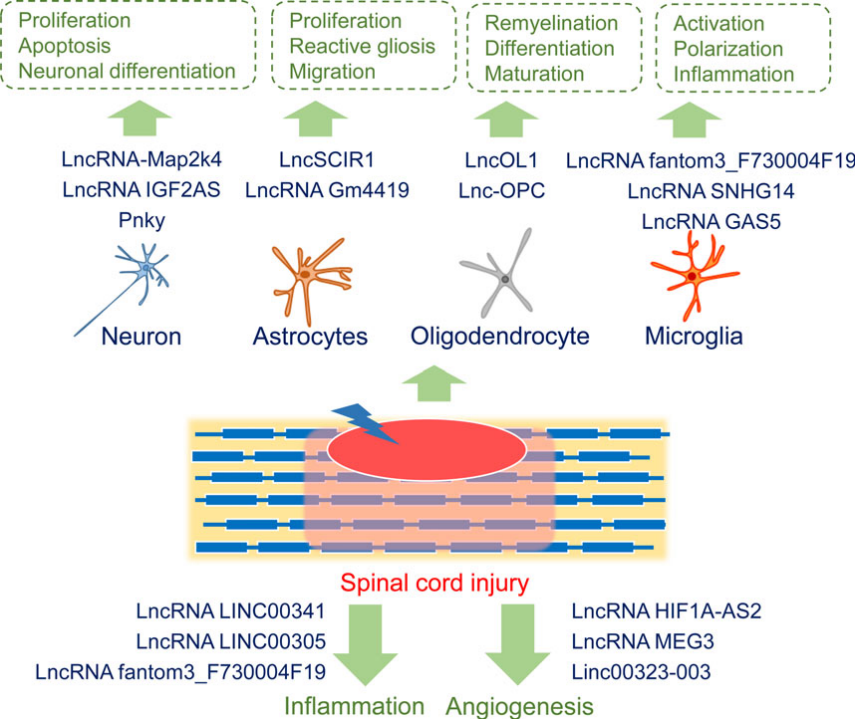
Involvement of lncRNAs in spinal cord injury.

SCI is a complex process integrating multiple related targets and signalling pathways in the nervous, immune and vascular systems, accompanied by many cellular and molecular mechanisms; however, the molecular mechanisms of SCI are not yet completely understood. Furthermore, no effective therapy is currently available to control the secondary injury following SCI 2, 92. Recent studies have found that the expression of lncRNAs changed after SCI and lncRNAs may play key roles in the pathological process of SCI. Utilization of lncRNAs provides an attractive proposition for the development of spinal cord repair. Furthermore, in contrast to miRNAs that are frequently expressed from various tissues and likely to target multiple mRNAs, however, lncRNAs display the characteristics of specificity 93. In the current review, we summarized the recent studies about lncRNAs in SCI, and we believed that this review will contribute to a global understanding of the molecular mechanisms of SCI and promote the development of clinical applications of lncRNAs (Fig. 1).

Moreover, several issues will be the research focus for further studies. First, the majority of the studies on the effect of lncRNAs on SCI are preclinical animal studies, and further investigations applying to humans will be needed in order to improve the transition to the clinic. Second, an appropriate delivery system is required for implementation of lncRNA therapy, so how to choose an appropriate vector which ensures the successful delivery of the lncRNAs to the desired targets is the future research direction. Furthermore, most of molecular mechanisms of lncRNAs in SCI are still unclear, so further investigations a deeper understanding of the causes and consequences of the dysregulation of lncRNAs in SCI and choosing the most promising lncRNAs will push the future development of lncRNAs for therapeutic applications, including the treatment of SCI.

## Conflict of interest

The authors have no conflict of interests to disclose.

## References

[1] Bareyre FM . Neuronal repair and replacement in spinal cord injury. J Neurol Sci. 2008; 265: 2055–2061. 10.1016/j.jns.2007.05.004 17568612

[2] Shi Z , Zhou H , Lu L , *et al* The roles of microRNAs in spinal cord injury. Int J Neurosci. 2017; 127: 1104–15. 28436759 10.1080/00207454.2017.1323208

[3] Singh A , Tetreault L , Kalsi‐Ryan S , *et al* Global prevalence and incidence of traumatic spinal cord injury. Clin Epidemiol. 2014; 6: 309–31. 25278785 10.2147/CLEP.S68889 PMC4179833

[4] Profyris C , Cheema SS , Zang D , *et al* Degenerative and regenerative mechanisms governing spinal cord injury. Neurobiol Dis. 2004; 15: 415–36. 15056450 10.1016/j.nbd.2003.11.015

[5] Kwon BK , Tetzlaff W , Grauer JN , *et al* Pathophysiology and pharmacologic treatment of acute spinal cord injury. Spine J. 2004; 4: 451–64. 15246307 10.1016/j.spinee.2003.07.007

[6] Tator CH . Update on the pathophysiology and pathology of acute spinal cord injury. Brain Pathol. 1995; 5: 407–13. 8974623 10.1111/j.1750-3639.1995.tb00619.x

[7] Di Gesualdo F , Capaccioli S , Lulli M . A pathophysiological view of the long non‐coding RNA world. Oncotarget. 2014; 5: 10976–96. 25428918 10.18632/oncotarget.2770 PMC4294373

[8] Guttman M , Rinn JL . Modular regulatory principles of large non‐coding RNAs. Nature. 2012; 482: 339–46. 22337053 10.1038/nature10887 PMC4197003

[9] Lam MT , Li W , Rosenfeld MG , *et al* Enhancer RNAs and regulated transcriptional programs. Trends Biochem Sci. 2014; 39: 170–82. 24674738 10.1016/j.tibs.2014.02.007 PMC4266492

[10] Mirza AH , Kaur S , Pociot F . Long non‐coding RNAs as novel players in beta cell function and type 1 diabetes. Hum Genomics. 2017; 11: 17. 28738846 10.1186/s40246-017-0113-7 PMC5525349

[11] Ulitsky I , Bartel DP . lincRNAs: genomics, evolution, and mechanisms. Cell. 2013; 154: 26–46. 23827673 10.1016/j.cell.2013.06.020 PMC3924787

[12] Bhatt DM , Pandya‐Jones A , Tong AJ , *et al* Transcript dynamics of proinflammatory genes revealed by sequence analysis of subcellular RNA fractions. Cell. 2012; 150: 279–90. 22817891 10.1016/j.cell.2012.05.043 PMC3405548

[13] Sun X , Haider Ali MSS , Moran M . The role of interactions of long non‐coding RNAs and heterogeneous nuclear ribonucleoproteins in regulating cellular functions. Biochem J. 2017; 474: 2925–35. 28801479 10.1042/BCJ20170280 PMC5553131

[14] Shi X , Sun M , Liu H , *et al* Long non‐coding RNAs: a new frontier in the study of human diseases. Cancer Lett. 2013; 339: 159–66. 23791884 10.1016/j.canlet.2013.06.013

[15] Evans JR , Feng FY , Chinnaiyan AM . The bright side of dark matter: lncRNAs in cancer. J Clin Investig. 2016; 126: 2775–82. 27479746 10.1172/JCI84421 PMC4966302

[16] Yang L , Wang H , Shen Q , *et al* Long non‐coding RNAs involved in autophagy regulation. Cell Death Dis. 2017; 8: e3073. 28981093 10.1038/cddis.2017.464 PMC5680586

[17] Shi C , Zhang L , Qin C . Long non‐coding RNAs in brain development, synaptic biology, and Alzheimer's disease. Brain Res Bull. 2017; 132: 160–9. 28347717 10.1016/j.brainresbull.2017.03.010

[18] Lorenzen JM , Thum T . Long noncoding RNAs in kidney and cardiovascular diseases. Nat Rev Nephrol. 2016; 12: 360–73. 27140855 10.1038/nrneph.2016.51

[19] Barriocanal M , Fortes P . Long non‐coding RNAs in hepatitis C virus‐infected cells. Front Microbiol. 2017; 8: 1833. 29033906 10.3389/fmicb.2017.01833 PMC5625025

[20] Guttman M , Amit I , Garber M , *et al* Chromatin signature reveals over a thousand highly conserved large non‐coding RNAs in mammals. Nature. 2009; 458: 223–7. 19182780 10.1038/nature07672 PMC2754849

[21] Shen S , Jiang H , Bei Y , *et al* Long non‐coding RNAs in cardiac remodeling. Cell Physiol Biochem. 2017; 41: 1830–7. 28376483 10.1159/000471913

[22] Niu ZS , Niu XJ , Wang WH . Long non‐coding RNAs in hepatocellular carcinoma: Potential roles and clinical implications. World J Gastroenterol. 2017; 23: 5860–74. 28932078 10.3748/wjg.v23.i32.5860 PMC5583571

[23] Costa FF . Non‐coding RNAs, epigenetics and complexity. Gene. 2008; 410: 9–17. 18226475 10.1016/j.gene.2007.12.008

[24] Rinn JL , Chang HY . Genome regulation by long noncoding RNAs. Annu Rev Biochem. 2012; 81: 145–66. 22663078 10.1146/annurev-biochem-051410-092902 PMC3858397

[25] Fan C , Tang Y , Wang J , *et al* Role of long non‐coding RNAs in glucose metabolism in cancer. Mol Cancer. 2017; 16: 130. 28738810 10.1186/s12943-017-0699-3 PMC5525357

[26] Xiong G , Feng M , Yang G , *et al* The underlying mechanisms of non‐coding RNAs in the chemoresistance of pancreatic cancer. Cancer Lett. 2017; 397: 94–102. 28254409 10.1016/j.canlet.2017.02.020

[27] Fang Y , Fullwood MJ . Roles, functions, and mechanisms of long non‐coding RNAs in cancer. Genomics Proteomics Bioinformatics. 2016; 14: 42–54. 26883671 10.1016/j.gpb.2015.09.006 PMC4792843

[28] Garitano‐Trojaola A , Agirre X , Prosper F , *et al* Long non‐coding RNAs in haematological malignancies. Int J Mol Sci. 2013; 14: 15386–422. 23887658 10.3390/ijms140815386 PMC3759866

[29] Li C , Chen J , Zhang K , *et al* Progress and prospects of long noncoding RNAs (lncRNAs) in hepatocellular carcinoma. Cell Physiol Biochem. 2015; 36: 423–34. 25968300 10.1159/000430109

[30] Chen S , Liang H , Yang H , *et al* Long non‐coding RNAs: the novel diagnostic biomarkers for leukemia. Environ Toxicol Pharmacol. 2017; 55: 81–6. 28841440 10.1016/j.etap.2017.08.014

[31] Thomson DW , Dinger ME . Endogenous microRNA sponges: evidence and controversy. Nat Rev Genet. 2016; 17: 272–83. 27040487 10.1038/nrg.2016.20

[32] Wang KC , Chang HY . Molecular mechanisms of long noncoding RNAs. Mol Cell. 2011; 43: 904–14. 21925379 10.1016/j.molcel.2011.08.018 PMC3199020

[33] Ding Y , Song Z , Liu J . Aberrant LncRNA expression profile in a contusion spinal cord injury mouse model. Biomed Res Int. 2016; 2016: 9249401. 27689092 10.1155/2016/9249401 PMC5027055

[34] Duran RC , Yan H , Zheng Y , *et al* The systematic analysis of coding and long non‐coding RNAs in the sub‐chronic and chronic stages of spinal cord injury. Sci Rep. 2017; 7: 41008. 28106101 10.1038/srep41008 PMC5247719

[35] Wang J , Hu B , Cao F , *et al* Down regulation of lncSCIR1 after spinal cord contusion injury in rat. Brain Res. 2015; 1624: 314–20. 26254726 10.1016/j.brainres.2015.07.052

[36] Yang H , Liu CC , Wang CY , *et al* Therapeutical strategies for spinal cord injury and a promising autologous astrocyte‐based therapy using efficient reprogramming techniques. Mol Neurobiol. 2016; 53: 2826–42. 25863960 10.1007/s12035-015-9157-7

[37] Li WC , Jiang DM , Hu N , *et al* Lipopolysaccharide preconditioning attenuates neuroapoptosis and improves functional recovery through activation of Nrf2 in traumatic spinal cord injury rats. Int J Neurosci. 2013; 123: 240–7. 23215850 10.3109/00207454.2012.755181

[38] Zhang M , Tao W , Yuan Z , *et al* Mst‐1 deficiency promotes post‐traumatic spinal motor neuron survival *via* enhancement of autophagy flux. J Neurochem. 2017; 143: 244–56. 28833175 10.1111/jnc.14154

[39] Lin N , Chang KY , Li Z , *et al* An evolutionarily conserved long noncoding RNA TUNA controls pluripotency and neural lineage commitment. Mol Cell. 2014; 53: 1005–19. 24530304 10.1016/j.molcel.2014.01.021 PMC4010157

[40] Ramos AD , Andersen RE , Liu SJ , *et al* The long noncoding RNA Pnky regulates neuronal differentiation of embryonic and postnatal neural stem cells. Cell Stem Cell. 2015; 16: 439–47. 25800779 10.1016/j.stem.2015.02.007 PMC4388801

[41] Lv HR . lncRNA‐Map2k4 sequesters miR‐199a to promote FGF1 expression and spinal cord neuron growth. Biochem Biophys Res Comm. 2017; 490: 948–54. 28655615 10.1016/j.bbrc.2017.06.145

[42] Zhang X , Chen K , Song C , *et al* Inhibition of long non‐coding RNA IGF2AS has profound effect on inducing neuronal growth and protecting local‐anaesthetic induced neurotoxicity in dorsal root ganglion neurons. Biomed Pharmacother. 2016; 82: 298–303. 27470366 10.1016/j.biopha.2016.04.042

[43] Lukovic D , Stojkovic M , Moreno‐Manzano V , *et al* Concise review: reactive astrocytes and stem cells in spinal cord injury: good guys or bad guys? Stem Cells. 2015; 33: 1036–41. 25728093 10.1002/stem.1959

[44] Zhang X , Yao H , Qian Q , *et al* Cerebral mast cells participate in postoperative cognitive dysfunction by promoting astrocyte activation. Cell Physiol Biochem. 2016; 40: 104–16. 27855371 10.1159/000452528

[45] Figley CR , Stroman PW . The role(s) of astrocytes and astrocyte activity in neurometabolism, neurovascular coupling, and the production of functional neuroimaging signals. Eur J Neuorsci. 2011; 33: 577–88. 10.1111/j.1460-9568.2010.07584.x 21314846

[46] Singh S , Joshi N . Astrocytes: inexplicable cells in neurodegeneration. Int J Neurosci. 2017; 127: 204–9. 27035359 10.3109/00207454.2016.1173692

[47] Chen X , Liu L , Qian R , *et al* Expression of Sam68 associates with neuronal apoptosis and reactive astrocytes after spinal cord injury. Cell Mol Neurobiol. 2017; 37: 487–98. 27236696 10.1007/s10571-016-0384-x PMC11482139

[48] White RE , Rao M , Gensel JC , *et al* Transforming growth factor alpha transforms astrocytes to a growth‐supportive phenotype after spinal cord injury. J Neurosci. 2011; 31: 15173–87. 22016551 10.1523/JNEUROSCI.3441-11.2011 PMC3213757

[49] Yu Y , Cao F , Ran Q , *et al* Long non‐coding RNA Gm4419 promotes trauma‐induced astrocyte apoptosis by targeting tumor necrosis factor alpha. Biochem Biophys Res Comm. 2017; 491: 478–85. 28688761 10.1016/j.bbrc.2017.07.021

[50] Khalaj AJ , Hasselmann J , Augello C , *et al* Nudging oligodendrocyte intrinsic signaling to remyelinate and repair: estrogen receptor ligand effects. J Steroid Biochem Mol Biol. 2016; 160: 43–52. 26776441 10.1016/j.jsbmb.2016.01.006 PMC5233753

[51] Waxman SG . Demyelination in spinal cord injury. J Neurol Sci. 1989; 91: 1–14. 2664092 10.1016/0022-510x(89)90072-5

[52] Mekhail M , Almazan G , Tabrizian M . Oligodendrocyte‐protection and remyelination post‐spinal cord injuries: a review. Prog Neurobiol. 2012; 96: 322–39. 22307058 10.1016/j.pneurobio.2012.01.008

[53] Kerr CL , Letzen BS , Hill CM , *et al* Efficient differentiation of human embryonic stem cells into oligodendrocyte progenitors for application in a rat contusion model of spinal cord injury. Int J Neurosci. 2010; 120: 305–13. 20374080 10.3109/00207450903585290

[54] Alizadeh A , Karimi‐Abdolrezaee S . Microenvironmental regulation of oligodendrocyte replacement and remyelination in spinal cord injury. J Physiol. 2016; 594: 3539–52. 26857216 10.1113/JP270895 PMC4929323

[55] He D , Wang J , Lu Y , *et al* lncRNA functional networks in oligodendrocytes reveal stage‐specific myelination control by an lncOL1/Suz12 complex in the CNS. Neuron. 2017; 93: 362–78. 28041882 10.1016/j.neuron.2016.11.044 PMC5600615

[56] Dong X , Chen K , Cuevas‐Diaz Duran R , *et al* Comprehensive identification of long non‐coding RNAs in purified cell types from the brain reveals functional LncRNA in OPC fate determination. PLoS Genet. 2015; 11: e1005669. 26683846 10.1371/journal.pgen.1005669 PMC4980008

[57] Shabab T , Khanabdali R , Moghadamtousi SZ , *et al* Neuroinflammation pathways: a general review. Int J Neurosci. 2017; 127: 624–33. 27412492 10.1080/00207454.2016.1212854

[58] Lawson LJ , Perry VH , Gordon S . Turnover of resident microglia in the normal adult mouse brain. Neuroscience. 1992; 48: 405–15. 1603325 10.1016/0306-4522(92)90500-2

[59] Yang X , Asakawa T , Han S , *et al* Neuroserpin protects rat neurons and microglia‐mediated inflammatory response against oxygen‐glucose deprivation‐ and reoxygenation treatments in an in vitro study. Cell Physiol Biochem. 2016; 38: 1472–82. 27035834 10.1159/000443089

[60] Wang HM , Zhang T , Huang JK , *et al* Edaravone attenuates the proinflammatory response in amyloid‐beta‐treated microglia by inhibiting NLRP3 inflammasome‐mediated IL‐1beta secretion. Cell Physiol Biochem. 2017; 43: 1113–25. 28977782 10.1159/000481753

[61] Liu G , Fan G , Guo G , *et al* FK506 attenuates the inflammation in rat spinal cord injury by inhibiting the activation of NF‐kappaB in microglia cells. Cell Mol Neurobiol. 2017; 37: 843–55. 27572744 10.1007/s10571-016-0422-8 PMC11482064

[62] Liu X , Huang S , Liu C , *et al* PPP1CC is associated with astrocyte and microglia proliferation after traumatic spinal cord injury in rats. Pathol Res Pract. 2017; 213: 355–1364. 10.1016/j.prp.2017.09.020 29033188

[63] Peng J , Wu Y , Tian X , *et al* High‐throughput sequencing and co‐expression network analysis of lncRNAs and mRNAs in early brain injury following experimental subarachnoid haemorrhage. Sci Rep. 2017; 7: 46577. 28417961 10.1038/srep46577 PMC5394545

[64] Qi X , Shao M , Sun H , *et al* Long non‐coding RNA SNHG14 promotes microglia activation by regulating miR‐145‐5p/PLA2G4A in cerebral infarction. Neuroscience. 2017; 348: 98–106. 28215748 10.1016/j.neuroscience.2017.02.002

[65] Gordon S . Alternative activation of macrophages. Nat Rev Immunol. 2003; 3: 23–35. 12511873 10.1038/nri978

[66] Cao L , He C . Polarization of macrophages and microglia in inflammatory demyelination. Neurosci Bull. 2013; 29: 189–98. 23558588 10.1007/s12264-013-1324-0 PMC5561884

[67] Franco R , Fernandez‐Suarez D . Alternatively activated microglia and macrophages in the central nervous system. Prog Neurobiol. 2015; 131: 65–86. 26067058 10.1016/j.pneurobio.2015.05.003

[68] Miron VE , Boyd A , Zhao JW , *et al* M2 microglia and macrophages drive oligodendrocyte differentiation during CNS remyelination. Nat Neurosci. 2013; 16: 1211–8. 23872599 10.1038/nn.3469 PMC3977045

[69] Sun D , Yu Z , Fang X , *et al* LncRNA GAS5 inhibits microglial M2 polarization and exacerbates demyelination. EMBO Rep. 2017; 18: 1801–16. 28808113 10.15252/embr.201643668 PMC5623836

[70] Hawthorne AL , Popovich PG . Emerging concepts in myeloid cell biology after spinal cord injury. Neurotherapeutics. 2011; 8: 252–61. 21400005 10.1007/s13311-011-0032-6 PMC3101835

[71] Neirinckx V , Coste C , Franzen R , *et al* Neutrophil contribution to spinal cord injury and repair. J Neuroinflammation. 2014; 11: 150. 25163400 10.1186/s12974-014-0150-2 PMC4174328

[72] Atianand MK , Fitzgerald KA . Long non‐coding RNAs and control of gene expression in the immune system. Trends Mol Med. 2014; 20: 623–31. 25262537 10.1016/j.molmed.2014.09.002 PMC4252818

[73] Geng H , Tan XD . Functional diversity of long non‐coding RNAs in immune regulation. Genes Dis. 2016; 3: 72–81. 27617274 10.1016/j.gendis.2016.01.004 PMC5013731

[74] Brazao TF , Johnson JS , Muller J , *et al* Long noncoding RNAs in B‐cell development and activation. Blood. 2016; 128: e10–9. 27381906 10.1182/blood-2015-11-680843 PMC5000579

[75] Aune TM , Crooke PS 3rd , Spurlock CF 3rd . Long noncoding RNAs in T lymphocytes. J Leukoc Biol. 2016; 99: 31–44. 26538526 10.1189/jlb.1RI0815-389R PMC6608032

[76] Panzeri I , Rossetti G , Abrignani S , *et al* Long intergenic non‐coding RNAs: novel drivers of human lymphocyte differentiation. Front Immunol. 2015; 6: 175. 25926836 10.3389/fimmu.2015.00175 PMC4397839

[77] Mirsafian H , Manda SS , Mitchell CJ , *et al* Long non‐coding RNA expression in primary human monocytes. Genomics. 2016; 108: 37–45. 26778813 10.1016/j.ygeno.2016.01.002

[78] Huang TS , Wang KC , Quon S , *et al* LINC00341 exerts an anti‐inflammatory effect on endothelial cells by repressing VCAM1. Physiol Genomics. 2017; 49: 339–45. 28500253 10.1152/physiolgenomics.00132.2016 PMC5538877

[79] Zhang DD , Wang WT , Xiong J , *et al* Long noncoding RNA LINC00305 promotes inflammation by activating the AHRR‐NF‐kappaB pathway in human monocytes. Sci Rep. 2017; 7: 46204. 28393844 10.1038/srep46204 PMC5385552

[80] Norenberg MD , Smith J , Marcillo A . The pathology of human spinal cord injury: defining the problems. J Neurotrauma. 2004; 21: 429–40. 15115592 10.1089/089771504323004575

[81] Kato H , Kanellopoulos GK , Matsuo S , *et al* Neuronal apoptosis and necrosis following spinal cord ischemia in the rat. Exp Neurol. 1997; 148: 464–74. 9417826 10.1006/exnr.1997.6707

[82] Loy DN , Crawford CH , Darnall JB , *et al* Temporal progression of angiogenesis and basal lamina deposition after contusive spinal cord injury in the adult rat. J Comp Neurol. 2002; 445: 308–24. 11920709 10.1002/cne.10168

[83] Holtz A , Nystrom B , Gerdin B . Relation between spinal cord blood flow and functional recovery after blocking weight‐induced spinal cord injury in rats. Neurosurgery. 1990; 26: 952–7. 2362673 10.1097/00006123-199006000-00005

[84] Dong R , Liu XQ , Zhang BB , *et al* Long non‐coding RNA‐CRNDE: a novel regulator of tumor growth and angiogenesis in hepatoblastoma. Oncotarget. 2017; 8: 42087–97. 28178668 10.18632/oncotarget.14992 PMC5522051

[85] Huang JK , Ma L , Song WH , *et al* LncRNA‐MALAT1 promotes angiogenesis of thyroid cancer by modulating tumor‐associated macrophage FGF2 protein secretion. J Cell Biochem. 2017; 118: 4821–30. 28543663 10.1002/jcb.26153

[86] Khorshidi A , Dhaliwal P , Yang BB . Noncoding RNAs in tumor angiogenesis. Adv Exp Med Biol. 2016; 927: 217–41. 27376737 10.1007/978-981-10-1498-7_8

[87] Zhou J , Huang H , Tong S , *et al* Overexpression of long non‐coding RNA cancer susceptibility 2 inhibits cell invasion and angiogenesis in gastric cancer. Mol Med Rep. 2017; 16: 5235–40. 28849111 10.3892/mmr.2017.7233 PMC5647078

[88] Li L , Wang M , Mei Z , *et al* lncRNAs HIF1A‐AS2 facilitates the up‐regulation of HIF‐1alpha by sponging to miR‐153‐3p whereby promoting angiogenesis in HUVECs in hypoxia. Biomed Pharmacother. 2017; 96: 165–72. 28985553 10.1016/j.biopha.2017.09.113

[89] He C , Yang W , Yang J , *et al* Long noncoding RNA MEG3 negatively regulates proliferation and angiogenesis in vascular endothelial cells. DNA Cell Biol. 2017; 36: 475–81. 28418724 10.1089/dna.2017.3682

[90] Liu J , Li Q , Zhang KS , *et al* Downregulation of the long Non‐Coding RNA Meg3 promotes angiogenesis after ischemic brain injury by activating notch signaling. Mol Neurobiol. 2016; 54: 8179–90. 27900677 10.1007/s12035-016-0270-z PMC5684256

[91] Fiedler J , Breckwoldt K , Remmele CW , *et al* Development of long noncoding RNA‐based strategies to modulate tissue vascularization. J Am Coll Cardiol. 2015; 66: 2005–15. 26516004 10.1016/j.jacc.2015.07.081 PMC4631810

[92] Silva NA , Sousa N , Reis RL , *et al* From basics to clinical: a comprehensive review on spinal cord injury. Prog Neurobiol. 2014; 114: 25–57. 24269804 10.1016/j.pneurobio.2013.11.002

[93] Yu B , Zhou S , Yi S , *et al* The regulatory roles of non‐coding RNAs in nerve injury and regeneration. Prog Neurobiol. 2015; 134: 122–39. 26432164 10.1016/j.pneurobio.2015.09.006

